# Extrapontine Myelinolysis-Induced Parkinsonism in a Patient with Adrenal Crisis

**DOI:** 10.1155/2012/327058

**Published:** 2012-12-17

**Authors:** Yahia Z. Imam, Maher Saqqur, Hassan Alhail, Dirk Deleu

**Affiliations:** ^1^Neurology Section, Department of Medicine, Hamad Medical Corporation, P.O. Box 3050, Doha, Qatar; ^2^Division of Neurology, University of Alberta, Edmonton, AB, Canada

## Abstract

*Background*. Extrapontine myelinolysis (EPM) has been well described in the presence of rapid correction of hyponatremia. It is seldom reported with adrenal insufficiency. We report a unique case where a patient developed EPM as a result of adrenal insufficiency where the brain MRI revealed symmetrical lesion in the basal ganglia with pallidal sparing. *Case Report*. A 30-year-old gentleman with panhypopituitarism developed adrenal crisis, hyponatremia, and hyponatremic encephalopathy. Seven days after the rapid correction of hyponatremia, he developed parkinsonism and neuropsychiatric symptoms. MRI showed extrapontine myelinolysis without central pontine myelinolysis. *Conclusion*. Extrapontine myelinolysis without central pontine myelinolysis is rare and should raise a concern of associated adrenal insufficiency in the right clinical setting. Rapid correction of hyponatremia particularly in steroid-deficient states should be avoided as it can predispose to extrapontine myelinolysis. Magnetic resonance imaging is very helpful in supporting the diagnosis of EPM.

## 1. Background

Central pontine myelinolysis (CPM) was first recognized in 1959 by Adams et al. [1]. In this paper; autopsy findings of myelin sheath destruction in a symmetrical fashion in the centre of the basis pontis were described. These lesions tend to spare the axons, the neuronal cell bodies, and the blood vessels with no signs of inflammation in the surrounding tissue. Malnutrition and alcohol consumption were the deemed causatives. Later on, the association was made with rapid correction of hyponatremia [2, 3]. Additionally liver disease, burns, and postliver transplantation were considered notorious culprits [2, 3].

It is now recognized that identical pathological demyelination to the ones seen in CPM can occur elsewhere, that is, extrapontine myelinolysis (EPM) either in combination with CPM or alone; collectively they were called osmotic demyelination [2, 3]. However, isolated EPM is relatively rare [2].

We presented a unique case of isolated EPM where a patient developed parkinsonism and neuropsychiatric symptoms 1 weeks after correction of hyponatremia in the setting of adrenal insufficiency. 

## 2. Case History

A 30-year-old man known to have panhypopituitarism on replacement therapy suffered an adrenal crisis characterized by fever, abdominal pain, and vomiting, following a tooth extraction. This resulted in a severe hyponatremia of 105 mmol/L and hyponatremic encephalopathy manifesting as confusion, agitation, and stupor for which he was admitted. Sepsis workup was negative. Magnetic resonance imaging (MRI) and cerebrospinal fluid exams were normal. Electroencephalogram (EEG) showed only diffuse bilateral slowing. Thyroid function tests were within normal limits and random cortisol level as well as a synacthen test confirmed the diagnosis of adrenal insufficiency.

**Figure 1:**
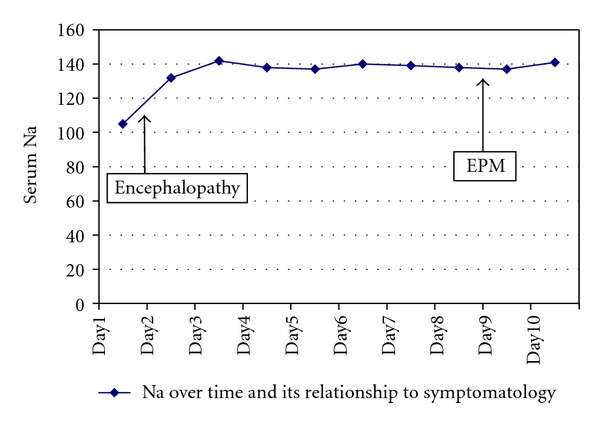
Serum Na by time.

 He was infused with normal saline to correct the hyponatremia as well as stress doses of hydrocortisone. After 72 hours his serum sodium level was 142 mmol/L (Figure 1). The patient's general condition improved over the next 2-3 days. On day 9 after admission, he began to deteriorate again with the development of slowness of speech and movement, emotional liability, and swallowing difficulties progressing to severe hypomimia, rigidity in the upper limbs, and spasticity in the lower limb. 

**Figure 2:**
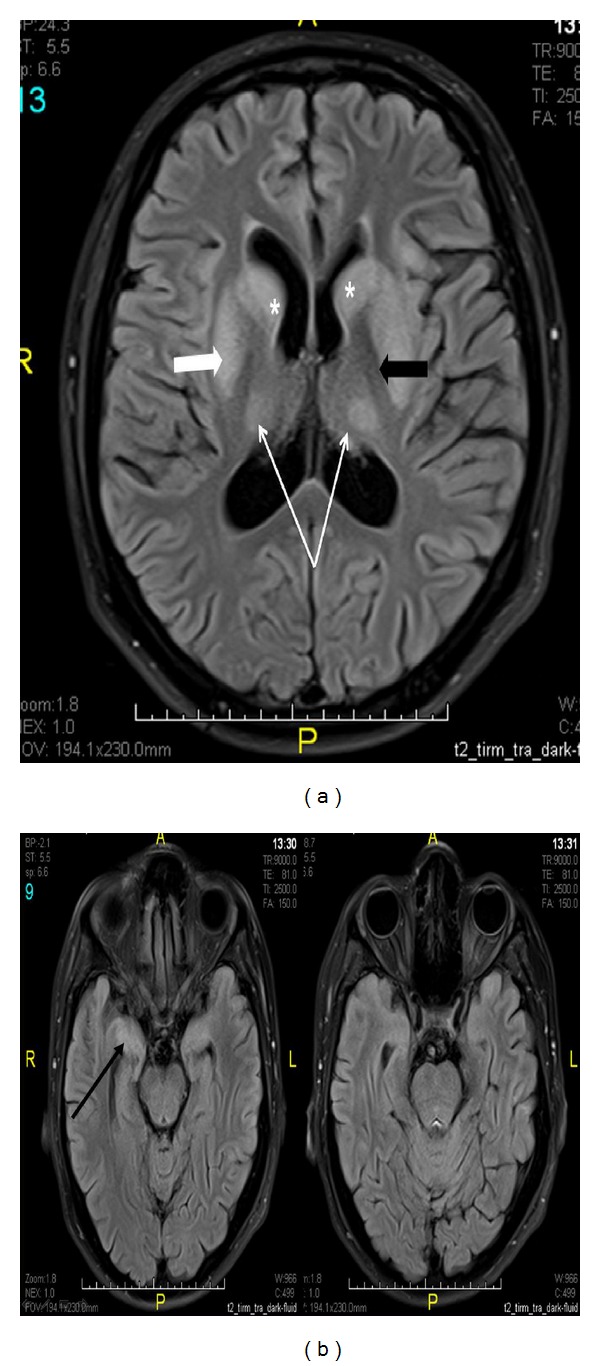
(a) MRI brain: axial FLAIR image showing hyperintense signal in the caudate nuclei bilaterally (asterisk marks), both putamina bilaterally (white thick arrow pointing to the right putamen), and both thalami (thin white arrows). Note the sparring of the globus pallidi (black thick arrow). (b) Axial FLAIR of the brain showing increased signal in the temporal lobes (namely, the anterior temporal lobes, particularly the hippocampi (black arrow)). The Pons show no sign of demyelination.

MRI revealed EPM without CPM (Figures 2(a) and 2(b)) affecting symmetrically the basal ganglia and thalami but sparing the globus pallidi. In addition, there was increased signal intensity in both hippocampal regions. 

To improve his parkinsonian syndrome he was empirically started on levodopa/carbidopa (125 mg tid) titrated up to control symptoms.

 Followup after 2 months showed moderate improvement. The patient regained most of the activities of daily living after being totally dependent. He was able to ambulate without assistance and his parkinsonian symptoms were under control with the help of medicine.

## 3. Discussion

In general parkinsonism, pseudobulbar symptoms, tetraparesis, and various movement disorders have been described with EPM [2, 4–9].

Our case report is unique for the following reasons. Firstly EPM without CPM is rare. Secondly, only 5 cases of EPM without CPM in association with adrenal insufficiency (Table 1) have been reported [4–9] and this is usually in the context of rapid correction of hyponatremia.

Typical MRI features of EPM include involvement of the cerebellum, the cerebral white matter, the basal ganglia (the most common site), and thalami, with sparing of the palladium [2, 9]. The lesions appear hyperintense on T2-weighted and FLAIR sequences and appear hypointense on T1 sequence [10, 11]. These findings alone are not specific for osmotic demyelination and must be interpreted in the appropriate clinical setting.

Treatment is usually symptomatically aimed at controlling parkinsonism, spasticity, and movement disorders and is often rewarding [4, 9].

Osmotic demyelination was originally regarded as carrying a grave prognosis with outcomes including death and severe disability. However, favourable outcomes are increasingly reported [9, 12, 13]. This is explained by the advancement of MRI in picking up the disease earlier in the course of the disease including the asymptomatic cases and the development of critical care services. However, MRI does not seem to predict prognosis or clinical improvement. The latter usually precedes radiological resolution if any [13].

In conclusion, EPM without CPM is rare and should raise a concern of associated adrenal insufficiency in the right clinical setting. Rapid correction of hyponatremia particularly in steroid deficient states should be avoided as it can predispose to EPM. A favourable prognosis is increasingly recognized and symptomatic treatment is the mainstay of management. MRI of the brain is very helpful in the diagnosis, but not so in terms of prognosis.

## Figures and Tables

**Table 1:** Cases in the literature with parkinsonism and isolated EPM with adrenal insufficiency.

Article	Cause of hyponatremia	Symptomatology	Outcome
Gujjar et al., 2010 [4]	Addison's disease and military tuberculosis (TB)	Parkinsonism	Good recovery
Al-Mamari et al., 2009 [5]	Addison's disease and miliary TB	Parkinsonism	Partial recovery
Srimanee et al., 2009 [6]	Hypopituitarism and secondary adrenal insufficiency	Dystonia	Not stated
Okada et al., 2005 [7]	Hypopituitarism and secondary adrenal insufficiency	Parkinsonism	Good recovery
Lasheen et al., 2005 [8]	Panhypopituitarism, pituitary microadenoma, and secondary adrenal insufficiency	Neuropsychiatric, dysarthria, and dystonia	Not stated
